# Hair Levels of Lead, Cadmium, Selenium, and Their Associations with Neurotoxicity and Hematological Biomarkers in Children from the Mojana Region, Colombia

**DOI:** 10.3390/molecules30153227

**Published:** 2025-08-01

**Authors:** Jenny Palomares-Bolaños, Jesus Olivero-Verbel, Karina Caballero-Gallardo

**Affiliations:** 1 Functional Toxicology Group, School of Pharmaceutical Sciences, Zaragocilla Campus, University of Cartagena, Cartagena 130014, Colombia; 2 Environmental and Computational Chemistry Group, School of Pharmaceutical Sciences, Zaragocilla Campus, University of Cartagena, Cartagena 130014, Colombia

**Keywords:** children’s health, pollution, metals, neurotoxicity, biomarkers

## Abstract

Heavy metals are a major toxicological concern due to their adverse effects on human health, particularly in children exposed to contaminated areas. This study evaluated biomarkers of exposure in 253 children aged 6 to 12 from Magangue, Achi, and Arjona (Bolivar, Colombia), analyzing their relationship with neurotoxicity and hematological markers. The mean Pb concentrations at the study sites were 1.98 µg/g (Magangue) > 1.51 µg/g (Achi) > 1.24 µg/g (Arjona). A similar pattern was observed for Cd concentrations for Magangue (0.39 µg/g) > Achi (0.36 µg/g) > Arjona (0.14 µg/g). In contrast, Se concentrations followed a different trend for Arjona (0.29 µg/g) > Magangue (0.21 µg/g) > Achi (0.16 µg/g). The proportion of Se/Pb molar ratios > 1 was higher in Arjona (3.8%) than in Magangue (0.9%) and Achi (2.0%). For Se/Cd ratios, values > 1 were also more frequent in Arjona (70.7%), exceeding 20% in the other two locations. Significant differences were found among locations in red and white blood cell parameters and platelet indices. Neurotransmitter-related biomarkers, including serotonin, monoamine oxidase A (MAO-A), and acetylcholinesterase levels, also varied by location. Principal component analysis showed that Pb and Cd had high loadings on the same component as PLT, WBC, and RDW, and while Se loaded together with HGB, PDW, MCHC, MCH, and MCV, suggesting distinct hematological patterns associated with each element. Multiple linear regression analysis demonstrated a statistically significant inverse association between hair Pb levels and serotonin concentrations. Although MAO-A and Cd showed negative β coefficients, these associations were not statistically significant after adjustment. These findings highlight the potential impact of toxic element exposure on key hematological and neurochemical parameters in children, suggesting early biological alterations that may compromise health and neurodevelopment.

## 1. Introduction

The study of environmental pollution as a determining factor in human health has gained increasing global attention, as it enables a deeper understanding of the exposure pathways and toxicological mechanisms of xenobiotics [1]. As a result, toxicological and epidemiological research has increasingly focused on children’s health and the impacts of chemical pollutants, with the aim of safeguarding their well-being and ensuring that environmental contamination, primarily caused by anthropogenic activities, does not compromise their health or optimal development [2]. Among environmental pollutants, lead (Pb) and cadmium (Cd) have been widely studied due to their increasing prevalence and high toxicity. Both elements have been classified by the World Health Organization (WHO) as chemicals of major public health concern and by the International Agency for Research on Cancer (IARC) as possible human carcinogens. The presence and bioavailability of these metallic elements in ecosystems are influenced by various environmental and contamination-related factors. Human activities such as mining, agriculture, oil extraction, and the smelting of metals and plastics are the primary sources of concern, as they release these metals into the environment in their most toxic forms [3].

The toxicity of heavy metals stems from their high chemical reactivity and distinctive physicochemical properties, which promote their accumulation in target organs and facilitate interactions with proteins, enzymes, and cellular receptors. These interactions can trigger a variety of toxicological effects—including neurotoxicity, immunotoxicity, hepatotoxicity, and carcinogenicity—depending on the route of exposure [4]. Lead compounds, in particular, can be readily absorbed through the digestive and respiratory tracts, cross the blood–brain barrier, and enter the plasma, thereby affecting the central nervous system. Additionally, Pb tends to accumulate in bone tissue before it is gradually excreted from the body [5]. The extent of absorption is influenced by individual metabolic factors and the presence of essential trace elements such as calcium (Ca), zinc (Zn), selenium (Se), copper (Cu), and iron (Fe) [6]. For this reason, determining Se concentrations in populations exposed to heavy metals may provide valuable insights into metal absorption and detoxification processes, and may also help assess its potential antagonistic role [7]. Although human studies are limited, the calculation of molar ratios has been recognized as a useful approach to estimate the antioxidant capacity of Se in relation to toxic metals such as Pb and Cd.

Currently, Pb and Cd are recognized as highly toxic metals with both carcinogenic and neurotoxic potential, posing a particular risk to children during fetal development and early cognitive stages. This vulnerability is due to their documented effects on neurotransmitter activity, even at low concentrations and across various chemical forms [8,9,10]. In terms of neurotoxicity, Pb is known to accumulate in the central nervous system (CNS) due to its high mobility. It crosses the blood–brain barrier primarily via divalent metal transporter 1 (DMT1) and calcium channels, disrupting intracellular calcium homeostasis—a process essential for neurotransmitter release and receptor activation during synaptic communication. Moreover, experimental studies in animal models have shown that Pb interacts with N-methyl-D-aspartate (NMDA) receptors, inducing multiple molecular alterations that contribute to neurological dysfunction [11].

Chronic exposure to Pb and Cd impairs neuronal function by disrupting monoaminergic neurotransmitters—including dopamine, noradrenaline, adrenaline, serotonin, and histamine—through the altered activity of monoamine oxidase A (MAO-A), an enzyme responsible for the metabolic degradation of these neurotransmitters [12]. Similarly, Cd, both individually and in combination with Pb and other heavy metals, negatively affects MAO-A activity and may interfere with serotonin metabolism in the nervous system. However, this pathway remains insufficiently explored in human studies and continues to be a key subject of investigation [13]. In addition, the interaction of these metals with neuronal calcium channels alters the release of the neurotransmitters GABA (gamma-aminobutyric acid) and glutamate. Pb also inhibits the enzymatic activity of acetylcholinesterase (AChE)—the enzyme responsible for hydrolyzing acetylcholine at the neuronal synapse—resulting in excitotoxicity within the cholinergic system and contributing to impaired cognitive development [14]. Due to their involvement in these pathways, AChE and MAO-A are considered potential biomarkers for detecting neurological dysfunction, alterations in serotonin levels, cholinergic imbalance, and for assessing their relationship with heavy metal-induced neurotoxicity [15].

The toxicological mechanisms of action of Pb and Cd in humans involve a wide range of metabolic, genetic, and biochemical alterations, including the inhibition of intracellular calcium signaling and enzymatic activity, particularly that of δ-aminolevulinic acid dehydratase (δ-ALAD). Inhibition of this enzyme results in elevated levels of δ-aminolevulinic acid (δ-ALA), a well-established biomarker of Pb exposure in human studies [16]. Both metals also induce cellular toxicity through mitochondrial dysfunction, inhibition of antioxidant enzymes, increased production of reactive oxygen species (ROS), and activation of apoptotic pathways [17]. At the enzymatic level, heavy metals such as Pb and Cd can impair ROS detoxification systems by reducing the activity of glutathione reductase, which catalyzes the conversion of oxidized glutathione (GSSG) to its reduced form (GSH) [6]. In recent years, Cd has been increasingly associated with neurotoxicity, genotoxicity, and pediatric cancers, primarily due to oxidative stress mechanisms capable of inducing DNA mutations, interfering with DNA repair processes, and altering gene expression [18,19].

Children’s increased toxicological susceptibility to metals such as Pb and Cd, which lack any known biological function, represents a significant public health concern. This heightened vulnerability is not only associated with adverse effects on the cognitive and functional development of the central nervous system but also with the metabolic interactions of these metals with essential elements such as Se. Moreover, the persistence of heavy metal toxicity over time from early childhood into adulthood further underscores its long-term impact. In the Bolivar Department of Colombia, many communities are subject to chronic environmental contamination. The study area, in particular, exhibits high levels of exposure to heavy metals due to activities such as deforestation, urbanization, agriculture, and artisanal mining, which contribute to the pollution of the Magdalena, Cauca, and San Jorge rivers. Elevated concentrations of these elements have been reported in fish, water, and sediments from these rivers [20]. Additionally, fishing and rice cultivation are common practices in the region, and the consumption of these products has been linked to Cd and As exposure in other parts of the world [21]. Mercury has also been detected in the hair of adults living along the Magdalena River, with concentrations exceeding established safety thresholds [22,23].

The primary concern in these communities is the potential health impact of heavy metal exposure on children residing in the region. Within this context, we hypothesize that environmental exposure to toxic elements such as lead (Pb) and cadmium (Cd) is associated with hematological alterations and neurotoxicity biomarkers in children, and that selenium (Se) may play a modulating role in these toxic effects. Therefore, the aim of this cross-sectional epidemiological study was to (1) determine the concentrations of Pb, Cd, and Se in hair samples of children aged 6 to 12 years residing in environmentally vulnerable areas of the Colombian Caribbean; (2) analyze the association between metal exposure and hematological and neurotoxicity biomarkers (AChE, MAO-A, and serotonin); and (3) evaluate the Se/Pb and Se/Cd molar ratios to explore the potential antagonistic effect of selenium on Pb- and Cd-induced toxicity.

## 2. Results

### 2.1. General Characteristics and Dietary Habits of the Study Population

Through informed consent and voluntary participation, a total of 253 hair and blood samples were collected from children residing in Magangue (n = 84), Achi (n = 80), and Arjona (n = 89). Information on general characteristics of the participants, including age, sex, and anthropometric measurements, is presented in Table 1. The mean age of all children was 9.1 ± 0.1 years, with a geometric mean of 8.9 years. No statistically significant differences were observed in the mean age among the three sites (*p* > 0.05).

Sex distribution was relatively uniform across the study locations, with an overall composition of 54.5% male and 45.5% female participants, and no statistically significant differences in sex distribution among the sites. Similarly, no significant differences were observed in weight, height, or body mass index (BMI) across the three locations. The mean BMI of the study population was 16.7 ± 0.2, falling below the normative thresholds defined by the Colombian Institute of Family Welfare (ICBF) growth and development standards.

Information on parental age, occupation, and consumption habits (tobacco and alcohol) of the parents is shown in Table 2. The mean age of the parents was 33.5 ± 0.3 years, with a GM of 33.1 years; no statistical significance was observed between sites. Parental occupations were categorized into agriculture, fishing, various occupations, and others (including commerce, bricklaying, and mechanics). In Magangue, 11.9% of parents were engaged in fishing, while in Achi, 15.0% worked in agriculture and only 2.5% in fishing. In the reference site (Arjona), 5.6% of parents were farmers, and none reported working in fishing, with the majority classified under the others category. A high proportion of parents reported alcohol consumption across all sites, with a significantly lower percentage in Achi (28.4%).

On the other hand, information on dietary habits was obtained for 70% of the children through a structured survey. The frequency of food consumption (meals/week) by site is shown in Table 3. The most commonly consumed foods were rice, bread, meat, eggs, and dairy products, while cereals, grains, and fruits were among the least consumed across all three locations. Fish consumption was higher in Magangue, where 20.0% of children reported more than 10 times per week, followed by tuber, with 9.1% reporting >10 meals/week. In contrast, the proportions of children with similarly high fish consumption were lower in Achi (4.3%) and Arjona (0.0%). Conversely, a higher percentage of children indicated consuming more than 10 meals per week of vegetables in Arjona (10.1%) and Achi (10.9%), compared to only 1.8% in Magangue. Additionally, significant differences in metal concentrations were observed according to the frequency of food consumption. Higher Pb levels were found in children who reported consuming meat and vegetables more than 10 times per week (*p*  <  0.05), while Cd concentrations were significantly elevated in those with frequent intake of fish and meat (*p*  <  0.05) (Supplementary Figure S3). These results suggest that dietary habits may be contributing to the internal exposure to toxic metals in this population.

### 2.2. Concentrations of Pb, Cd, and Se in Hair

**Figure 1:**
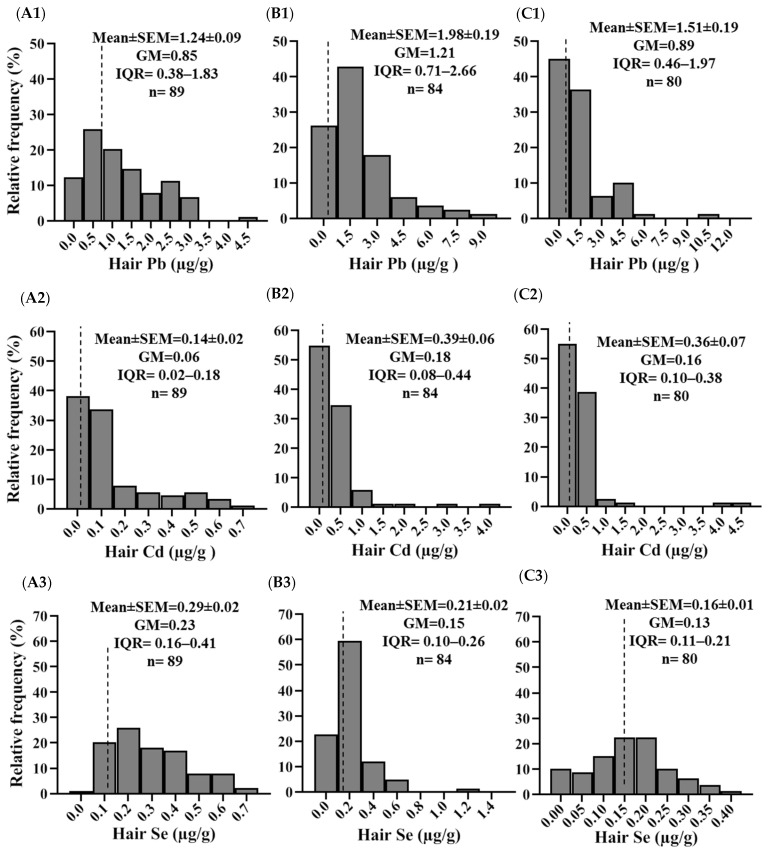
Frequency distribution and concentrations of Pb, Cd, and Se in hair samples from (**A1**–**A3**) Arjona, (**B1**–**B3**) Magangue, and (**C1**–**C3**) Achi. Error bars represent the standard error of the mean (SEM). GM: geometric mean and IQR: interquartile range (25th–75th percentile).

**Figure 2:**
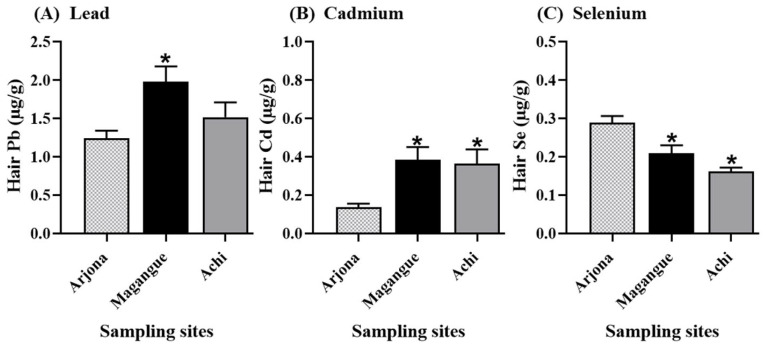
Comparison of the concentrations of the measured elements among the three study sites: (**A**) Lead, (**B**) Cadmium, and (**C**) Selenium. *. Indicate statistically significant differences compared to the reference site (*p* < 0.05).

The frequency distributions of total concentrations of Pb, Cd, and Se in hair are presented in Figure 1. The geometric mean concentrations of these elements in Magangue and Achi followed the order Se > Cd > Pb, whereas in Arjona, the order was Cd > Se > Pb. The mean concentrations of Pb were highest in Magangue (1.98 µg/g), followed by Achi (1.51 µg/g) and Arjona (1.24 µg/g). A similar pattern was observed for Cd, Magangue (0.39 µg/g) > Achi (0.36 µg/g) > Arjona (0.14 µg/g). In contrast, Se concentrations followed a different trend: Arjona (0.29 µg/g) > Magangue (0.21 µg/g) > Achi (0.16 µg/g). Children residing in Magangue and Achi had significantly higher mean concentrations of Cd and Pb in hair compared to those in Arjona (*p* < 0.05), while Se levels were significantly lower than those observed in the reference site (*p* < 0.05) (Figure 2).

The mean concentrations of Pb, Cd, and Se in hair, stratified by sex, are presented in Supplementary Figure S1. No statistically significant differences were found in Pb and Se concentrations between males and females in Magangue, Achi, and Arjona. However, Cd levels were significantly higher in females than in males in both Achi and Arjona (*p* < 0.05). The correlation matrix between the concentrations of Pb, Cd, Se, and the children’s anthropometric variables is presented in Supplementary Table S1. The results show a significant positive association between Pb and Cd levels, and an inverse correlation between Pb and Se. No significant associations were found between metal concentrations and weight or height (Supplementary Figure S2).

The frequencies of Se concentrations by site and sex are presented in Table 4. According to international classification standards, 57% of participants were classified as selenium deficient. The highest percentages of Se-deficient children (<0.2 µg/g) were observed in Magangue and Achi, with a significantly greater proportion of males than females affected. In contrast, children in Arjona more frequently presented sufficient Se levels. No cases of Se excess were identified in any of the study sites.

**Figure 3:**
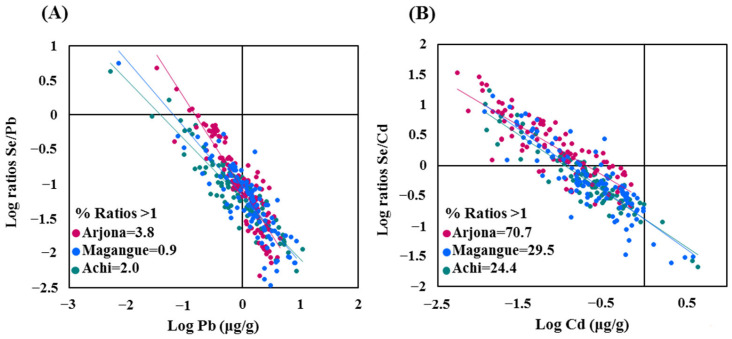
Scatter plot showing the relationship between the logarithm of Pb, Cd, and Se levels (µg/g) in hair and the logarithmic molar ratios of Se/Pb (**A**) and Se/Cd (**B**).

The molar ratios of Se to Pb and Cd are presented in the scatter plot of the logarithm of Pb and Cd levels and the logarithm of the Se/metal molar ratio in hair (Figure 3). The percentage of Se/Pb molar ratios greater than 1 in hair samples was 0.9 in Magangue and 2.0 in Achi. Meanwhile, the Se/Cd molar ratios are higher, reaching 29.5% in Magangue and 24.4% in Achi. In the reference site (Arjona), the proportions of the Se/Pb and Se/Cd molar ratios greater than 1 were notably higher, at 3.8% and 70.7%, respectively.

**Figure 4:**
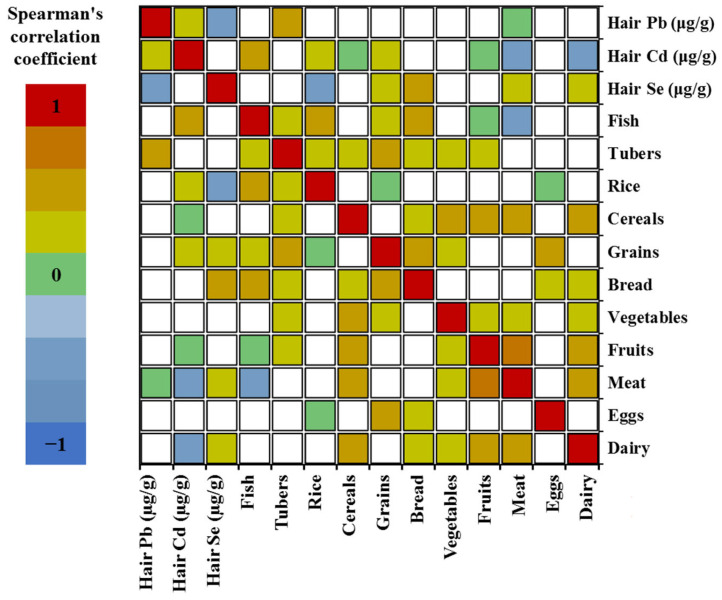
Spearman correlation matrix between the frequency of food consumption (meals/week) and the concentrations of Pb, Cd, and Se (µg/g) in children’s hair.

The Spearman correlation matrix between the concentrations of Pb, Cd, and Se (µg/g) in the hair and of frequency of food consumption (meals/week) in the overall population is shown in Figure 4. The results revealed a significant but weak positive correlation between Pb levels and tuber consumption (ρ = 0.203; *p* = 0.01), between Cd concentrations and fish consumption (ρ = 0.209; *p* = 0.01) and rice (ρ = 0.171; *p* = 0.03). Conversely, Pb levels were negatively correlated with meat consumption (ρ = −0.170; *p* = 0.03), while Cd concentrations showed inverse correlations with cereal (ρ = −0.175; *p* = 0.02), meat (ρ = −0.229; *p* = <0.01), and dairy product (ρ = −0.221; *p* < 0.01). Se levels were positively correlated with the consumption of bread (ρ = 0.278; *p* < 0.01) and dairy products (ρ = 0.170; *p* = 0.03), but showed a negative correlation with rice consumption (ρ = −0.223; *p* < 0.01). Detailed ρ and *p* values are provided in Supplementary Table S2.

### 2.3. Hematological Parameters

The results of hematological variables and the frequency (%) of values outside the different sampling sites are presented in Table 5. Several statistically significant differences (*p* < 0.05) were observed in mean values among children from Magangue, Achi, and Arjona. Specifically, significant differences were found in red blood cell parameters (HGB, MCH, MCHC, and RDW), white blood cell indices (WBC, LYM, GRA, LYM%, and GRA%), and platelet parameters (MPV and PDW). A higher percentage of children in Magangue presented with low hematocrit (HTC) and lymphocyte (LYM) levels (9.5% and 14.3%, respectively), and with MCHC values above the reference range (21.4%). In Achi, 8.8% and 10.0% of children showed low HGB, HTC, and GRA values, respectively, while elevated values were observed in RDW (11.3%), WBC (12.5%), and GRA (11.3%). In Arjona, abnormal values were also observed in several variables, including HTC, MCV, LYM, GRA, LYM%, and GRA%.

**Figure 5:**
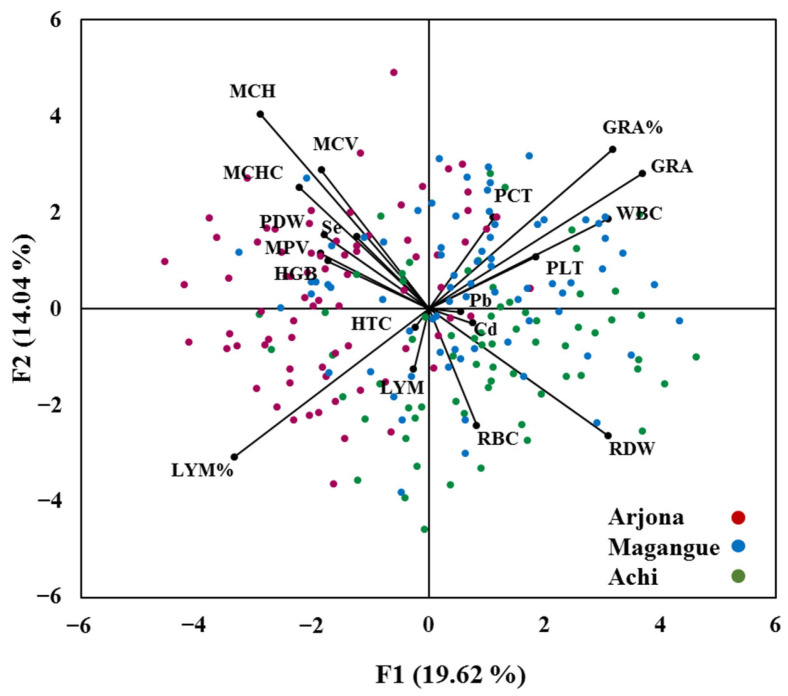
PCA biplots and scree plots showing the relationship between hematological variables and metal concentrations (Pb, Cd, Se) across the study population.

Principal component analysis (PCA) was performed to assess potential interactions between hematological parameters and metal concentrations, as shown in Supplementary Table S3. A total of five components were extracted, explaining 67.6% of the total variance. Factors 1 and 2 accounted for 33.6% of the variance (Figure 5). Factor 1 exhibited strong positive loadings for WBC, RDW, GRA, and GRA%, along with negative loadings for MCH and LYM%. In Factor 2, MCH showed a strong positive loading. Visual inspection of the scatter plots and biplots of Factor 1 and Factor 2 scores revealed associations between Pb, Cd, and PLT, WBC, and RDW, as well as between Se and HGB, PDW, MCHC, MCH, and MCV. Moreover, some overlapping clusters were observed, particularly between Magangue and Achi. Additionally, in the overall child population, cadmium concentration was positively correlated with WBC, LYM, and GRA, and negatively correlated with RBC, HGB, MCHC, MPV, and PLT.

### 2.4. Biochemical Markers

**Figure 6:**
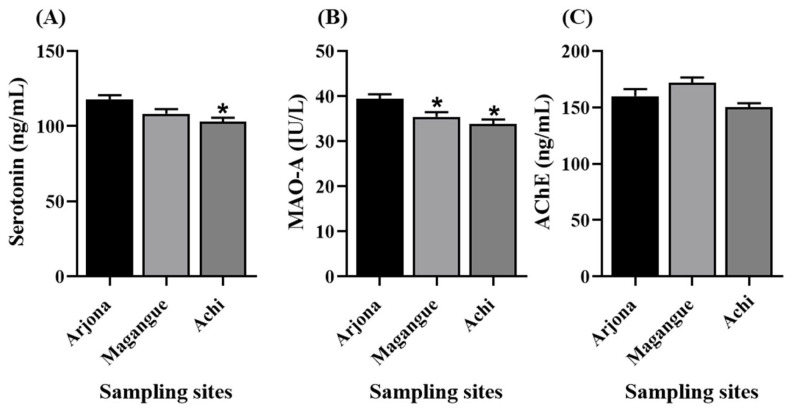
Blood levels of (**A**) serotonin, (**B**) monoamine oxidase A (MAO-A), and (**C**) acetylcholinesterase (AChE) across the study sites. Data are expressed as mean ± SEM. * Significant difference compared to Arjona (*p* < 0.05).

The mean concentrations of biochemical markers in blood showed notable differences between the study sites (Figure 6). Serotonin levels were highest in children from Arjona (117.6 ± 3.1 ng/mL), followed by Magangue (108.3 ± 3.1 ng/mL), and Achi (102.8 ± 2.8 ng/mL). Similarly, the mean activity of MAO-A was 39.4 ± 1.0 IU/L in Arjona, 35.4 ± 1.0 IU/L in Magangue, and 33.8 ± 1.0 IU/L in Achi. For AChE, mean values were 159.9 ± 6.2 ng/mL in Arjona, 171.6 ± 4.9 ng/mL in Magangue, and 150.0 ± 3.7 ng/mL in Achi. Statistical analysis revealed significant differences in these biochemical markers, particularly between the reference site (Arjona) and the other two locations.

Spearman correlation analysis indicated a positive correlation between hair Cd concentrations and blood serotonin (ρ = 0.189; *p* = 0.04) and AChE (ρ = 0.211; *p* = 0.03) levels in children from Magangue. In addition, a negative correlation was found between hair Pb and blood serotonin levels in children from Achi (ρ = −0.201; *p* = 0.03). In Arjona, Pb and Cd concentrations in hair were negatively correlated with Serotonin (ρ = −0.232; *p* = 0.02 and ρ = −0.216; *p* = 0.02, respectively), and Cd was also negatively correlated with AChE (ρ = −0.337; *p* < 0.01). Additionally, in the total population, hair Cd levels showed a negative correlation with MAO-A (ρ = −0.112; *p* = 0.04). Conversely, hair Se concentrations were positively correlated with blood serotonin and MAO-A levels and negatively correlated with AChE. In the case of serotonin, 40.5, 47.5, and 28.1% of the children from Magangue, Achi, and Arjona, respectively, exhibited levels below the reference range (101–283 ng/mL). Furthermore, both serotonin and MAO-A levels were positively associated with hematological parameters such as HGB, MCH, and MCHC.

Hematological and biochemical variables that showed a correlation with Pb, Cd, and Se concentrations were further analyzed using multiple linear regression, with results presented in Table 5. A significant positive association was obtained between Pb concentration and WBC count (β = 0.193; *p* = 0.043). Likewise, RDW was positively associated with Cd concentration. In contrast, Se levels showed significant positive association with serotonin (β = 24.20; *p* = 0.025), MAO-A (β = 7.617; *p* = 0.038), HGB (β = 0.792; *p* = 0.015), MCV (β = 3.472; *p* = 0.028), MCH (β = 2.288; *p* = 0.001), and MCHC (β = 1.441; *p* = 0.014). Conversely, an inverse association was found between serotonin levels and Pb concentrations (β = −2.575; *p* = 0.022), as well as between Cd and both MAO-A levels (β = −2.279; *p* = 0.048) and MCHC (β = −0.381; *p* = 0.035). In addition, Se levels were inversely associated with RDW (β = −1.204; *p* = 0.004). To refine these associations, the regression model was adjusted for potential confounders, including age, sex, height, and weight. After adjustment, the inverse associations between serotonin and Pb and between MCHC and Cd remained significant, as did the positive associations between Se levels and serotonin, HGB, MCV, and MCH (Table 6).

## 3. Discussion

Human exposure to toxic elements is strongly associated with pollution from anthropogenic activities such as mining, industry, and agriculture, which release these substances into air, soil, and water sources [25,26]. Once in the environment, they can accumulate in sediments or undergo microbial transformations that promote their biomagnification through the food chain until reaching human consumption. This exposure route is well-documented in the scientific literature [27]. The concentrations of Pb and Cd detected in children’s hair samples in this study suggest ongoing environmental exposure affecting communities in the Bolívar Department.

Previous environmental monitoring studies have reported high levels of pollution in this region, primarily linked to deforestation, urbanization, agriculture, and artisanal mining—activities that significantly affect the Magdalena, Cauca, and San Jorge rivers. Elevated concentrations of mercury (Hg) have been detected in fish, water, and sediments from these aquatic ecosystems [20,28]. These areas also support fishing and rice cultivation, products whose consumption has been associated with increased levels of Cd and As in human populations in other regions [21]. Furthermore, biomonitoring studies have reported Hg concentrations in human hair that exceed permissible safety thresholds in populations residing along the Magdalena River [22,23,29]. Together, these findings indicate multiple sources of environmental contamination in the study area and support the present evidence of Pb and Cd exposure in children.

Factors such as age, sex, and developmental stage may influence metal concentrations in children [30], in addition to prenatal and postnatal exposures [31], which are critical variables when evaluating bioaccumulation and toxicity mechanisms. Variations in exposure levels may be related to differences in behavior, recreational activities, and anatomical characteristics typical of childhood. In particular, sex has been associated with differential absorption, distribution, metabolism, and toxicological responses to environmental elements [32]. In the present study, adjustments were made for potential confounding variables, including age, sex, weight, and height. Notably, sex-based differences in Pb and Cd concentrations were observed, with higher levels detected in females (Figure S2).

Children’s dietary habits were among the factors evaluated, revealing that the most frequently consumed foods across the study population were rice, bread, meat, eggs, and dairy products, while cereals, legumes, and fruits were the least consumed. Overall, correlations between the frequency of food consumption and hair concentrations of Pb, Cd, and Se were weak. Nevertheless, a weak but notable positive correlation was observed between Cd levels and the consumption frequency of fish and rice. Conversely, a negative correlation was identified between selenium concentrations in hair and the frequency of rice consumption.

Lead is commonly present in the environment as a result of anthropogenic activities such as mining, fishing, and smelting. In children, exposure can occur through multiple routes, including inhalation and both direct and indirect ingestion. In the present study, higher Pb concentrations were observed in children from Magangue and Achi compared to the reference site, with significantly elevated levels detected in Magangue (Figure 1 and Figure 2). Notably, 26% of the children assessed had hair Pb concentrations exceeding the reference threshold of 2 µg/g. These site-specific differences may be associated with parental occupational activities, as a greater proportion of parents in Magangue reported working in fishing, a finding that aligns with the higher frequency of fish consumption indicated in dietary surveys (Table 2 and Table 3). These concentrations exceed those previously reported by Manjarres-Suarez et al. [33], who assessed Pb and other trace elements in adolescents from a Caribbean community in Bolivar. The elevated Pb levels identified in this study are of significant concern, as even low concentrations in children are associated with high neurotoxic risk. Lead exposure during early life has been linked to cognitive deficits and reduced IQ, primarily due to its interference with glutamatergic and cholinergic signaling, calcium homeostasis, mitochondrial function, reactive oxygen species (ROS) generation, and neurotransmitter activity [34]. Currently, there is no universally established reference value for Pb levels in hair; however, given that Pb is a non-essential and highly toxic element, its presence in children’s bodies should ideally be absent.

In the case of Cd, this element is present in various environmental matrices and can enter the human body through ingestion, inhalation, or indirect exposure, particularly via the consumption of contaminated food such as shellfish and fish [18]. In this study, low Cd concentrations were detected in hair samples; however, significantly higher levels were observed in children from Magangue and Achi compared to the reference site (Figure 2). This pattern may be associated with the elevated rice consumption reported in these populations, which showed a positive correlation with Cd concentrations. The presence of Cd in rice and its potential toxicological impact have been widely documented, particularly following the Itai-Itai disease outbreak in Japan, attributed to chronic ingestion of Cd-contaminated rice. As a result, maximum permissible limits have been established for rice-based products intended for human consumption. Although studies involving children remain limited, the Cd levels found in this study exceed those previously reported in adult populations [35,36,37].

It is important to highlight that even low levels of Cd may pose a potential health risk for children at the physiological level. Cadmium can substitute for zinc in biological systems, inhibit DNA repair mechanisms, and induce carcinogenic mutations. It may also interfere with the activity of DNA methyltransferases, resulting in either hypermethylation or hypomethylation depending on the exposure level [38]. Moreover, in vitro and in vivo studies in genetics and molecular biology have demonstrated Cd’s involvement in neurotoxic processes due to its association with severe neurological disorders such as Parkinson’s disease, Alzheimer’s disease, amyotrophic lateral sclerosis (ALS), and stroke, which may be triggered by chronic low-level environmental exposure. Cd has also been identified as an endocrine disruptor with potential transgenerational effects [39].

From a pediatric immunotoxicology perspective, recent studies have shown that the presence of elements such as Cd and Pb in the body can induce significant alterations in hematological parameters, even at levels considered low [10]. These elements may interfere with the normal function of blood cells, making hematological biomarkers valuable tools for assessing both health status and exposure to environmental contaminants in children. Parameters such as HGB, HCT, RBC, WBC, MCV, MCH, and MCHC are commonly used to diagnose conditions like anemia, a prevalent childhood disorder typically associated with nutritional deficiencies [40]. However, from a toxicological standpoint, anemia may also result from heavy metal exposure, which can impair erythrocyte and hemoglobin production and function [41,42].

In this study, statistical analyses revealed negative correlations between HGB and MCHC with Cd and Pb concentrations. Conversely, positive correlations were observed between these metals and immune parameters such as WBC, PLT, PCT, and LYM. Notably, multiple linear regression analysis showed a significant positive association between WBC and Pb, as well as between RDW and Cd, alongside an inverse association between MCHC and Cd. These associations may be attributed to the ability of Cd and Pb to alter immune function by either activating or impairing leukocytes and lymphocytes [43]. Given that WBCs play a central role in both innate and adaptive immune responses, any alteration in their function could compromise the immune system’s capacity to respond adequately to infections or inflammatory stimuli. Moreover, several studies have suggested that exposure to these metals during critical developmental stages may lead to persistent immunotoxic effects, potentially increasing the risk of chronic or autoimmune diseases later in life [44].

Furthermore, the neurotoxic mechanisms of Pb suggest that it accumulates in the body due to its high affinity for the central nervous system (CNS). Its mobility enables it to cross the blood–brain barrier via divalent metal transporter 1 (DMT1) and calcium channels, disrupting intracellular calcium homeostasis—a process essential for neurotransmitter release and receptor activation during synaptic communication [12]. Based on this mechanism, the present study evaluated serotonin and MAO-A levels, revealing that the lowest values were observed in children with the highest concentrations of Pb and Cd. These associations remained statistically significant even after adjusting for confounding variables. An inverse association was also found between serotonin levels and Pb concentration. These findings are consistent with previous studies reporting functional and structural damage to the nervous system caused by Pb, including decreased serotonin levels [13]. Additionally, in vivo studies have shown a negative correlation between Pb exposure and MAO-A activity [45,46].

Cadmium neurotoxicity has been associated with the blockade of ion channels and altered neurotransmitter release, particularly serotonin, which may contribute to neurodegenerative processes during critical developmental stages [47,48]. Additionally, in vitro studies have shown that Pb inhibits the activity of the enzyme AChE, responsible for acetylcholine hydrolysis at the synaptic cleft. This inhibition can lead to excitotoxicity in the cholinergic system, adversely affecting cognitive development and neurological performance in children [14]. Although a correlation between serotonin and AChE was observed in this study, the adjusted linear regression model did not reveal a statistically significant association. In contrast, Se maintained a positive association with both serotonin and MAO-A levels, highlighting its potential protective role in modulating neurotransmitter balance.

Nevertheless, the descriptive data reveal a downward trend in AChE levels among children from Achi compared to those from the reference site (Arjona), even though the difference was not statistically significant (Figure 6A). These findings suggest that cholinergic pathways may be subtly affected in populations chronically exposed to Pb and Cd. Given that AChE is essential for the termination of synaptic transmission, its inhibition by heavy metals, even at low levels, may indicate early neurochemical disruption. This aligns with the significant decreases observed in serotonin and MAO-A (Figure 6B,C), reinforcing the hypothesis of an altered neurochemical balance in exposed children.

The study measured Se concentrations in children’s hair to assess its potential antagonistic role against Pb and Cd toxicity, considering its function in detoxification processes. The results revealed that Se/Pb and Se/Cd molar ratios exceeded 1 in only a minority of cases, indicating a high prevalence of Se deficiency. This contrasts with findings from other studies [7], raising doubts about the protective effect of Se in this population and suggesting an elevated toxicological risk due to the limited availability of this essential micronutrient.

The Se deficiency observed in children may be attributed to multiple interrelated factors. One key aspect is the low Se content in the soils of the study areas, which limits its concentration in local agricultural products, particularly in rice, identified as one of the most frequently consumed foods according to dietary surveys [49,50]. Additionally, children’s diets in rural environments tend to lack diversity and are often deficient in high-quality Se sources [51], largely due to socioeconomic conditions characterized by elevated poverty levels, limited access to nutritious foods, and the absence of nutritional supplementation programs. Moreover, the presence of Pb and Cd may interfere with the bioavailability and metabolic utilization of essential micronutrients such as Se. These toxic elements can promote the formation of insoluble complexes with Se, thereby reducing its physiological availability, particularly under conditions of chronic exposure [52].

From a clinical and nutritional standpoint, Se is essential for the optimal function of the endogenous antioxidant defense system, primarily due to its role as a structural component of enzymes such as glutathione peroxidase and thioredoxin reductase. These enzymes protect cells against oxidative damage caused by ROS, which are commonly generated during heavy metal exposure [53,54]. In children, persistent Se deficiency has been associated with disruptions in neurological, immune, and endocrine development, as well as with increased vulnerability to infections and chronic inflammatory conditions [55,56]. In particular, an adequate Se/Pb and Se/Cd molar ratio has been proposed as a modulator of the cytotoxic and genotoxic effects of these metals. Se may act as a cofactor in DNA repair mechanisms and help attenuate lipid peroxidation in critical tissues such as the brain and liver [15,57].

In contexts of chronic environmental exposure, such as the populations evaluated in this study, insufficient Se intake not only weakens antioxidant defenses but may also exacerbate the neurotoxic and hematotoxic effects of toxic elements, particularly during critical periods of child development [58]. These findings highlight the urgent need to implement nutritional intervention strategies in exposed communities, with an emphasis on improving selenium status through dietary diversification or targeted supplementation, as a complementary measure to mitigate the cumulative toxicity of Pb and Cd in children [59]. Additionally, longitudinal studies are warranted to assess the modulatory role of Se across different physiological systems affected by these elements, including its potential application as a biomarker of metabolic resilience to toxic insult [60].

The findings of this study have important implications for public health, child nutrition, and environmental management. The detection of Pb and Cd in children’s hair underscores the need to establish continuous epidemiological surveillance programs, particularly in environmentally vulnerable regions. Moreover, the observed associations support the potential use of hematological and biochemical markers such as HGB, serotonin, and MAO-A activity as early clinical indicators of neurotoxic effects induced by these elements. From a nutritional perspective, the identification of limited intake of Se-rich foods highlights the urgency of implementing food education strategies that promote protective dietary habits against environmental toxicity. It also reinforces the need for public policies aimed at regulating environmental contamination and controlling agricultural practices such as the use of fertilizers that contribute to cadmium accumulation in staple foods like rice. Finally, the results contribute to the toxicological evidence of the multisystemic effects of chronic, low-level exposure to toxic elements and suggest the potential of micronutrient-based interventions as a complementary strategy to mitigate adverse health outcomes.

## 4. Materials and Methods

### 4.1. Study Area

**Figure 7:**
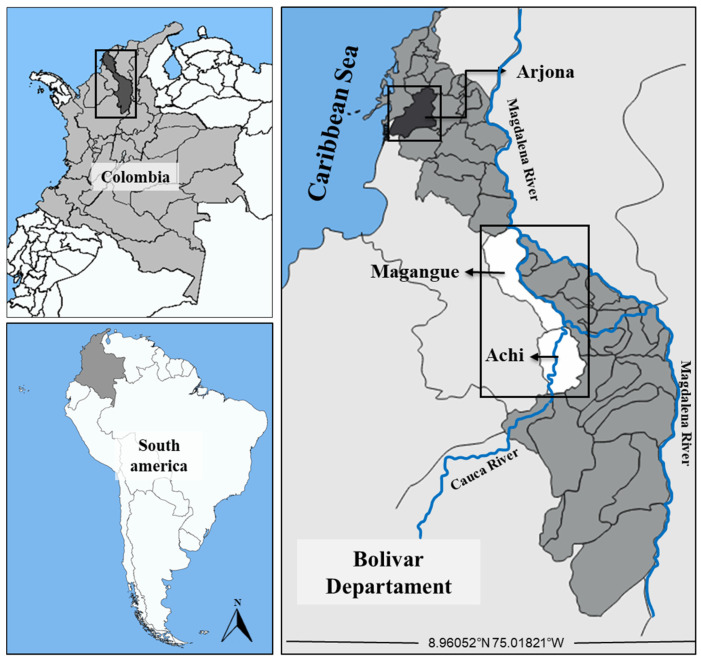
Geographical location of the study area in the Mojana Bolivarense region, showing the sampling sites in the municipalities of Magangue, Achi, and Arjona (Bolivar, Colombia).

This study was conducted in the Mojana Bolivarense, a subregion of the Bolivar department covering an area of 6.143 km^2^ and a total of 126.765 inhabitants. It comprises the municipalities of Achi, Magangue, Montecristo, Pinillos, San Jacinto, and Tiquisio, which are characterized by a high biodiversity of natural resources [61]. This economic and social development zone (ZODES) is primarily sustained by mining, agriculture, and aquaculture, supported by the presence of the Magdalena River, one of Colombia’s most significant water bodies. Over the years, various studies have evidenced contamination dynamics associated with these economic activities, suggesting that local populations are chronically exposed to toxic substances, including potentially hazardous elements. Sampling was conducted in the municipalities of Magangue (9°14′48″ N; 74°45′34″ W), Achi (8°34′09″ N; 74°33′22″ W), and Arjona (Figure 7), all located in rural territories along the Magdalena and Cauca rivers. Magangue is one of the most densely populated municipalities in the department and is known for the cultivation of both permanent and transient crops such as corn, yams, and especially, rice [62]. Achi, located along the Cauca River on the border with the Sucre department in the Momposina depression, has a hydrographic richness that supports intensive agricultural and fishing practices [63]. Both municipalities report higher fish and rice consumption rates compared to other areas in the Mojana Bolivarense [23,64]. Arjona was selected as a reference site due to its similar sociodemographic, climatic, and geological characteristics, and the absence of previous reports on environmental contamination by toxic elements. These conditions facilitate the interpretation of the results and ensure an appropriate comparison for the fulfillment of the study objectives.

### 4.2. Participant Selection and Data Collection

The study was conducted between January 2021 and February 2022, involving 253 children (both male and female), aged 6 to 12 years, residing in the municipalities of Magangue, Achi, and Arjona in the department of Bolívar. The participants were children of local fishermen, farmers, and miners, recruited through community outreach and socialization activities carried out with the support of municipal leaders. In all cases, participation was voluntary and authorized through informed consent signed by parents or guardians and assent by the children. The study protocol was approved by the Ethics Committee of the University of Cartagena (CE-119, 2019), in accordance with international standards and national bioethical regulations for research involving human subjects, in particular, the principles outlined in the Declaration of Helsinki. The sample size was estimated based on population projection provided by the National Administrative Department of Statistics (DANE), using the Z^2^ statistic, with a 9% margin of error and assuming a 50% probability of occurrence and non-occurrence of the event. After selection, a survey was administered to collect sociodemographic data, dietary habits, and anthropometric measurements, including weight, height, and body mass index (BMI).

### 4.3. Collection of Hair and Blood Samples

Blood samples were collected by venipuncture using anticoagulant-free, RNA-free tubes (Applied Biosystems, Foster City, CA, USA), under aseptic conditions with sterile, pyrogen-free materials, maintaining a closed system in accordance with Article 40 of Decree 1571 of 1993. All procedures complied with the scientific, technical, and administrative standards for health research established in Resolution 008430 of 1993 by the Ministry of Health and Social Protection. Samples were stored at −80 °C until analysis for enzymatic determinations. Hair samples were also collected for analysis of Cd, Pb, and Se concentrations. Approximately 100 mg of hair was obtained from the occipital region of the children and stored in white paper envelopes until digestion and pre-treatment, following previously established protocols. To ensure homogenization, the hair samples were cut into 1 mm fragments using sterilized scissors and transferred to 15 mL Falcon tubes. A standardized washing procedure was applied, involving sequential rinses with 2% Triton X-100, ultrapure deionized water, and acetone, followed by centrifugation cycles to eliminate external contaminants and interfering substances. The cleaned samples were dried in an oven at 40 °C for 12 h and stored in desiccators until analysis [65].

### 4.4. Analysis of Lead, Cadmium, and Selenium in Hair

The concentrations of the elements were measured using the optimized analytical method 7010 by atomic absorption spectroscopy (AAS) in human hair samples. The samples were digested with nitric acid (HNO_3_) and hydrogen peroxide (H_2_O_2_) following microwave-assisted acid digestion (EPA Method 3052) in a closed-vessel microwave system (Milestone STAR D Sorisole Bergamo, Italy) [66]. Once the quality control and optimization of the method were performed, the digested solutions were analyzed using a graphite furnace atomic absorption spectrometer (GFAAS) for Cd, Pb, and Se. Standard solutions (Merck, Darmstadt, Germany) were used for calibration, with nickel and magnesium nitrates as matrix modifiers, element-specific electrothermal cuvettes, and 0.2% nitric acid as the diluent. All measurements were performed in triplicate, and only data with a relative standard deviation (RSD) below 10% were considered.

### 4.5. Quality Control

To standardize the procedure, the digestion and analysis method was validated using the certified reference material (CRM), TORT-2 (National Research Council Canada, Ottawa, ON, Canada), evaluating parameters such as accuracy, precision, linearity, working range, limit of detection (LOD), and limit of quantification (LOQ). The validation considered appropriate sample weighting, temperature ramp protocols, and absorbance readings as provided by the iCE 3000 AA equipment (Thermo Fisher Scientific, Waltham, MA, USA). Linearity was assessed within the 0–20 µg/L range, with an acceptable correlation coefficient (R^2^ > 0.99). A sample weight of 100 mg was used. The LODs for Pb, Cd, and Se were 0.1, 0.02, and 0.03 µg/L, respectively. Recovery percentages exceeded 98%, and the relative standard deviation (RSD) was consistently below 10%. Analyses were performed using a graphite furnace atomic absorption spectrometer iCE 3000 AA (Thermo Fisher Scientific, Waltham, MA, USA), operating with the linear least squares fitting method and reporting results in µg/L. Both standard and coated graphite cuvettes were used, with temperature ramps ranging from 100 °C to 2500 °C, optimized according to each element.

### 4.6. Determination of Se/Toxic Metal Molar Ratios

Molar ratios between Se and toxic elements (Pb and Cd) were calculated using the formula: CSe*MSe/Ct*Mt, where CSe represents the concentration of Se in children’s hair and MSe its atomic mass. Ct and Mt refer to the concentration and atomic mass of the toxic elements (Cd or Pb), respectively. Ratios below 1 were interpreted as an indicator of limited detoxification potential, based on previous studies (Cd and Pb) [7].

### 4.7. Measurement of Biochemical Markers

To evaluate the neurological condition of the children, biochemical markers related to neurotransmission in the cholinergic and dopaminergic systems were measured, including acetylcholinesterase (AChE), monoamine oxidase A (MAO-A), and serotonin. The enzymatic activity of MAO-A was determined using the commercial ELISA kit Human monoamine oxidase MAO (CSBE-E10144h, CUSABIO BIOTECH, Wuhan, China), following the manufacturer’s instructions. Serotonin levels were quantified by ELISA method using the Enzyme Immunoassay for Quantification in Plasma, Serum, Platelets, and Human Urine (IBL International GMBH, Hamburg, Germany: No. RE 59121). The activity of AChE was measured using the colorimetric assay kit ab138871 (Abcam, Cambridge, MA, UK), which quantifies the production of thiocholine resulting from AChE activity. Absorbance readings were performed using a Multiskan^®^ Spectrum microplate spectrophotometer (Thermo Scientific, Barrington, IL, USA) at a wavelength of 450 nm.

### 4.8. Hematological Analysis

Hematological parameters were assessed using an automated impedance-based hemogram to evaluate red blood cell (RBC) indices (red blood cell count [RBC], hemoglobin [HGB], hematocrit [HCT], mean corpuscular volume [MCV], mean corpuscular hemoglobin [MCH], and mean corpuscular hemoglobin concentration [MCHC], as well as red cell distribution width [RDW]); white blood cell (WBC) indices (total white blood cell count [WBC], absolute lymphocyte count [LYM], absolute granulocyte count [GRA], lymphocyte percentage [LYM%], and granulocyte percentage [GRA%]); and platelet indices (platelet count [PLT], plateletcrit [PCT], mean platelet volume [MPV], and platelet distribution width [PDW]). Analyses were performed using the Abacus 380 Automated Hematology Analyzer (Diatron, Budapest, Hungary), with calibrations and quality control verified using reference blood samples, as described by [29].

### 4.9. Statistical Analysis

Quantitative variables were described using mean and standard error (SE). Data normality was evaluated using the Kolmogorov–Smirnov test, and homoscedasticity was assessed using Bartlett’s test. Variables that did not meet normality assumptions were transformed using the natural logarithm to approximate a normal distribution. Comparisons of means among the three study populations were performed using the Kruskal–Wallis test, followed by Dunn’s post hoc test for pairwise comparisons. Bivariate relationships between quantitative variables were assessed using Spearman’s correlation coefficient. Associations involving categorical variables—particularly those derived from sociodemographic and dietary data—were analyzed using the chi-squared (χ^2^) test. To explore multivariate relationships, two statistical approaches were employed: multiple linear regression (MLR) and principal component analysis (PCA). The MLR model included adjustments for potential confounding variables such as age, sex, weight, height, and sampling location. Model assumptions were verified through diagnostic procedures: normality of residuals was checked using Q–Q plots, homoscedasticity was assessed using residual-versus-fitted plots, and independence of observations was examined using residuals-versus-order plots. Multicollinearity was ruled out with a Variance Inflation Factor (VIF) < 10. All statistical tests were two-tailed, and a *p*-value < 0.05 was considered statistically significant. Data processing and analyses were conducted using Microsoft Excel 2016, GraphPad Prism 8, and IBM SPSS software version 20.

## 5. Conclusions

The exposure of children to heavy metals represents a significant public health concern, particularly given the heightened vulnerability associated with early developmental stages. The findings of this study underscore the need for ongoing biomonitoring in pediatric populations living in environmentally at-risk areas. The concentrations of Pb and Cd detected in hair samples reflect chronic exposure and validate the utility of hair as a non-invasive biological matrix for assessing long-term exposure to toxic metals in children. This approach facilitates the correlation between metal accumulation and potential health effects without the ethical and practical challenges associated with more invasive methods. Additionally, the integration of sociodemographic characterization highlights the role of poverty, malnutrition, and food insecurity as compounding risk factors that may exacerbate the toxicological burden in these communities. These factors must be considered when designing public health interventions. Neurotoxicity biomarkers, including serotonin and MAO-A levels, revealed significant alterations associated with low-level Pb and Cd exposure, indicating potential impacts on neurodevelopment. These results reinforce the importance of further research focused on long-term neurological, hematological, and nutritional consequences of environmental metal exposure in children.

## Figures and Tables

**Table 1:** General characteristics of children by sampling site.

Variable	Measurement	Total n = 253	Sampling Sites			Statistic	*p*-Value
			Arjona n = 89	Magangue n = 84	Achi = 80		
Age (years)	Mean ± SEM	9.1 ± 0.1	8.8 ± 0.2	9.5 ± 0.2	9.1 ± 0.2	KW = 5.3	0.07
	GM	8.9	8.6	9.3	8.9		
Sex	Male	138 (54.5)	43 (48.3)	43 (51.2)	52 (65.0)	*χ*^2^ = 5.9	0.05
	Female	115 (45.5)	46 (51.7)	41 (48.8)	28 (35.0)		
Weight (kg)	Mean ± SEM	30.6± 0.6	31.3 ± 0.9	29.2 ± 0.8	31.2 ± 1.0	KW = 2.4	0.29
	GM	29.4	29.9	28.3	30.0		
Height (cm)	Mean ± SEM	134.2 ± 0.8	135.9 ± 1.4	132.9 ± 1.3	133.6 ± 1.3	KW = 2.5	0.29
	GM	133.6	135.2	132.4	133.1		
BMI (kg/m^2^)	Mean ± SEM	16.7 ± 0.2	16.7 ± 0.3	16.3 ± 0.2	17.2 ± 0.3	KW = 3.0	0.22
	GM	16.5	16.4	16.1	16.9		

Data are presented as the mean ± standard error of the mean (SEM) or as frequencies expressed as number of data (percentage), n (%), depending on the variable. GM: geometric mean; KW: Kruskal–Wallis test followed by Dunn’s multiple comparison test; *χ*^2^ = Chi-square test.

**Table 2:** General characteristics of parents by sampling site.

Variable	Measurement		Study Area			Statistic	*p*-Value
		Total n = 253	Arjona n = 89	Magangue n = 84	Achi = 80		
Age (years)	Mother Mean ± SEM GM	33.6 ± 0.4 33.1	34.6 ± 0.7 34.0	33.0 ± 0.7 32.4	33.3 ± 0.6 32.8	KW = 3.2	0.21
	Father Mean ± SEM GM	38.9 ± 0.5 38.1	38.0 ± 0.7 37.4	38.9 ± 1.0 37.9	40.0 ± 0.9 39.2	KW = 1.9	0.38
Father occupation	Agriculture	17 (6.7)	5 (5.6)	0 (0.0)	12 (15.0)	*χ*^2^ = 46.1	<0.01 *
	Fishing	12 (4.7)	0 (0.0)	10 (11.9)	2 (2.5)		
	Various occupations	97 (38.3)	25 (28.1)	30 (35.7)	42 (52.5)		
	Others (commerce, bricklaying, mechanics)	127 (50.3)	59 (66.3)	44 (52.4)	24 (30.0)		
Alcohol consumption Father and mother	Yes	210 (44.8)	89 (51.4)	77 (54.6)	44 (28.4)	*χ*^2^ = 25.5	<0.01 *
	No	259 (55.2)	84 (48.6)	64 (45.4)	111 (71.6)		
Tobacco consumption Father and mother	Yes	19 (4.1)	11 (6.4)	7 (5.0)	1 (0.6)	*χ*^2^ = 7.3	0.03 *
	No	450 (95.9)	162 (93.6)	134 (95.0)	154 (99.4)		

Data are presented as the mean ± standard error of the mean (SEM) or as frequencies expressed as number of data (percentage), n (%), depending on the variable. GM: geometric mean; KW: Kruskal–Wallis test followed by Dunn’s multiple comparison test; *χ*^2^ = Chi-square test. *. Indicate statistical significance (*p* < 0.05).

**Table 3:** Food consumption of children by sampling site.

Variable	Category	Sampling Sites				Statistic *χ*^2^	*p*-Value
		Total n = 180	Arjona n = 79	Magangue n = 55	Achi = 46		
Fish (meals/week)	0–4	146 (81.1)	79 (100.0)	27 (49.1)	40 (87.0)	61.9	<0.01 *
	5–10	21 (11.7)	0 (0.0)	17 (30.9)	4 (8.7)		
	>10	13 (7.2)	0 (0.0)	11 (20.0)	2 (4.3)		
Tubers (meals/week)	0–4	118 (65.6)	50 (63.3)	35 (63.6)	33 (71.7)	7.1	0.13
	5–10	53 (29.4)	28 (35.4)	15 (27.3)	10 (21.7)		
	>10	9 (5.0)	1 (1.3)	5 (9.1)	3 (6.5)		
Rice (meals/week)	0–4	6 (3.3)	3 (3.8)	3 (5.5)	0 (0.0)	67.6	<0.01 *
	5–10	74 (41.1)	57 (72.2)	7 (12.7)	10 (21.7)		
	>10	100 (55.6)	19 (24.0)	45 (81.8)	36 (78.3)		
Cereal grains (Corn, wheat) (meals/week)	0–4	164 (91.1)	71 (89.9)	49 (89.1)	44 (95.7)	1.57	0.46
	5–10	16 (8.9)	8 (10.1)	6 (10.9)	2 (4.3)		
	>10	0 (0.0)	0 (0.0)	0 (0.0)	0 (0.0)		
Grains (lentils, beans) (meals/week)	0–4	171 (95.0)	79 (100.0)	53 (96.4)	39 (84.8)	16.0	<0.01 *
	5–10	9 (5.0)	0 (0.0)	2 (3.6)	7 (15.2)		
	>10	0 (0.0)	0 (0.0)	0 (0.0)	0 (0.0)		
Bread (meals/week)	0–4	89 (48.4)	40 (50.6)	16 (29.1)	33 (71.7)	19.5	<0.01 *
	5–10	77 (42.8)	32 (40.5)	34 (61.8)	11 (23.9)		
	>10	14 (7.8)	7 (8.9)	5 (9.1)	2 (4.3)		
Vegetables (meals/week)	0–4	115 (63.9)	51 (64.6)	37 (67.3)	27 (58.7)	4.2	0.38
	5–10	51 (28.3)	20 (25.3)	17 (30.9)	14 (30.4)		
	>10	14 (7.8)	8 (10.1)	1 (1.8)	5 (10.9)		
Fruits (meals/week)	0–4	121 (67.2)	43 (54.4)	49 (89.1)	29 (63.0)	20.2	<0.01 *
	5–10	57 (31.7)	35 (44.3)	5 (9.1)	17 (37.0)		
	>10	2 (1.1)	1 (1.3)	1 (1.8)	0 (0.0)		
Meat (meals/week)	0–4	107 (59.4)	30 (37.9)	51 (92.7)	26 (56.5)	46.4	<0.01 *
	5–10	61 (33.9)	42 (53.2)	4 (7.3)	15 (32.6)		
	>10	12 (6.7)	7 (8.9)	0 (0.0)	5 (10.9)		
Eggs (meals/week)	0–4	111 (61.7)	48 (60.8)	34 (61.8)	29 (63.0)	1.75	0.81
	5–10	63 (35.0)	29 (36.7)	18 (32.7)	16 (34.8)		
	>10	6 (3.3)	2 (2.5)	3 (5.5)	1 (2.2)		
Dairy products (meals/week)	0–4	99 (55.0)	29 (36.7)	38 (69.1)	32 (69.6)	17.9	<0.01 *
	5–10	79 (43.9)	49 (62.0)	17 (30.9)	13 (28.3)		
	>10	2 (1.1)	1 (1.3)	0 (0.0)	1 (2.2)		

Data are presented as frequencies n (%). Comparisons were performed using the Chi-square test (*χ*^2^), with the Monte Carlo method and 1000 simulations used to estimate *p*-values. *. Indicate statistical significance (*p* < 0.05).

**Table 4:** Classification of Se levels in children by site and sex, expressed as number of data (n) and percentage (%).

Selenium Level (µg/g) *	Classification	Arjona n = 89		Magangue n = 84		Achi n = 80	
		Male n (%)	Female n (%)	Male n (%)	Female n (%)	Male n (%)	Female n (%)
<0.2	Se-deficient	16 (18.0)	20 (22.5)	29 (34.5)	21 (25.0)	37 (46.2)	20 (25.0)
0.2–0.25	Se-marginal	1 (1.1)	5 (5.6)	3(3.6)	6 (7.1)	5 (6.3)	4 (5.0)
0.25–0.5	Se-sufficient	20 (22.5)	13 (14.6)	9 (10.7)	11 (13.1)	10 (12.5)	4 (5.0)
0.5–3	Se-rich	6 (6.7)	8 (9.0)	2 (2.4)	3 (3.6)	0 (0.0)	0 (0.0)
>3	Se-excessive	0 (0.0)	0 (0.0)	0 (0.0)	0 (0.0)	0 (0.0)	0 (0.0)

* Reference values for Se were taken from Zheng et al. [24].

**Table 5:** Hematologic parameters and frequency of abnormal values in blood samples from children, by sampling site.

Variable	Unit	Reference Value	Arjona n = 89			Magangue n = 84			Achi n = 80		
			Mean ± SEM	Below Reference (%)	Above Reference (%)	Mean ± SEM	Below Reference (%)	Above Reference (%)	Mean ± SEM	Below Reference (%)	Above Reference (%)
Red blood cell											
RBC	10^12^/L	4.1–5.6	4.7 ± 0.04	1.1	0.0	4.7 ± 0.05	0.0	0.0	4.7 ± 0.04	2.5	0.0
HGB	g/dL	11.5–16.3	13.3 ± 0.1	2.2	0.0	12.8 ± 0.1 *	2.4	0.0	12.4 ± 0.1 *	8.8	0.0
HTC	%	34.4–48.3	37.7 ± 0.3	9.0	0.0	37.5 ± 0.3	9.5	1.2	37.3 ± 0.2	8.8	0.0
MCV	fL	74.3–93	79.9 ± 0.5	10.1	0.0	80.4± 0.3	4.8	0.0	80.1 ± 0.5	6.3	0.0
MCH	pg	24.3–31.7	28.3 ± 0.2	2.2	0.0	27.6 ± 0.2 *	1.2	0.0	26.6 ± 0.2 *	6.3	1.3
MCHC	g/dL	31.9–35.1	35.4 ± 0.1	3.4	0.0	34.4 ± 0.1 *	1.2	21.4	33.2 ± 0.1 *	1.3	1.3
RDW	%	11.7–18.3	16.0 ± 0.1	0.0	0.0	16.9 ± 0.1 *	0.0	5.9	17.3 ± 0.1 *	0.0	11.3
White blood cell											
WBC	10^9^/L	3.7–11.9	6.6 ± 0.2	0.0	0.0	8.5 ± 0.2 *	0.0	5.9	9.1 ± 0.3 *	0.0	12.5
LYM	10^9^/L	2.2–6.8	2.9 ± 0.1	18.0	1.1	3.0 ± 0.1	14.3	0.0	3.2 ± 0.1 *	3.8	2.5
GRA	10^9^/L	2.5–7.5	3.4 ± 0.1	23.6	0.0	4.9 ± 0.2 *	2.4	5.9	4.8 ± 0.2 *	10.0	11.3
LYM	%	20–56.6	43.4 ± 1.2	1.1	18.0	35.6 ± 0.9 *	2.4	0.0	37.3 ± 1.0 *	1.3	1.3
GRA	%	33.2–74.7	49.5 ± 1.2	11.2	1.1	57.9 ± 1.0 *	0.0	3.6	52.2 ± 1.1	2.5	1.3
Platelet											
PLT	10^9^/L	194–477	294.9 ± 5.9	2.2	0.0	313.6 ± 8.0	5.9	2.4	285.1 ± 6.9	5.0	0.0
PCT	%	0.1–0.5	0.2 ± 0.01	0.0	0.0	0.2 ± 0.01	0.0	0.0	0.2 ± 0.01	1.3	0.0
MPV	fL	6.6–9.9	8.2 ± 0.1	0.0	1.1	7.8 ± 0.1 *	3.6	2.4	7.9 ± 0.1 *	3.8	3.8
PDW	%	25–50	38.7 ± 0.2	0.0	0.0	38.2 ± 0.2	0.0	0.0	38.1 ± 0.2 *	0.0	0.0

* Statistical significance (*p* < 0.05).

**Table 6:** Multiple linear regression analysis of Pb, Cd, and Se concentrations in hair and in relation to hematological and biochemical variables.

Variables	Hair Pb (µg/g)				Hair Cd (µg/g)				Hair Se (µg/g)			
	Not Adjusted		Adjusted		Not Adjusted		Adjusted		Not Adjusted		Adjusted	
	β (*p* Value)	95% CI	β (*p*-Value)	95% CI	β (*p*-Value)	95% CI	β (*p*-Value)	95% CI	β (*p*-Value)	95% CI	β (*p*-Value)	95% CI
Serotonin	−2.575 (0.022) *	−4.775; −0.375	−2.495 (0.042) *	−5.021; −0.031	−5.337 (0.100)	−11.71; 1.035	−4.867 (0.179)	−12.00; 2.263	24.20 (0.025) *	2.929; 45.48	24.78 (0.027) *	2.846; 46.72
MAO-A	−0.416 (0.275)	−1.166; 0.333	−0.372 (0.343)	−1.144; 0.399	−2.279 (0.048) *	−4.534; −0.024	−1.584 (0.177)	−3.885; 0.717	7.617 (0.038) *	0.424; 14.81	6.647 (0.073)	−0.645; 13.94
AChE	0.851 (0.652)	−2.859; 4.561	0.529 (0.787)	−3.319; 4.376	1.979 (0.709)	−8.485; 12.44	0.808 (0.884)	−10.08; 11.69	−21.67 (0.222)	−56.60; 13.26	−21.24 (0.245)	−57.21; 14.73
WBC	0.193 (0.043) *	−0.003; 0.389	0.174 (0.086)	−0.025; 0.373	0.206 (0.489)	−0.380; 0.722	0.161 (0.597)	−0.439; 0.761	−1.393 (0.150)	−3.297; 0.510	−1.125 (0.248)	−3.041; 0.790
HGB	−0.032 (0.341)	−0.099; 0.034	−0.026 (0.448)	−0.092; 0.041	−0.157 (0.118)	−0.354; 0.040	−0.159 (0.114)	−0.357; 0.038	0.792 (0.015) *	0.154; 1.431	0.739 (0.021) *	0.109; 1.370
MCV	−0.115 (0.483)	−0.438; 0.208	−0.067 (0.683)	−0.390; 0.256	0.579 (0.234)	−0.377; 1.536	0.664 (0.176)	−0.300; 1.629	3.472 (0.028) *	0.376; 6.568	3.302 (0.035) *	0.233; 6.371
MCH	−0.012 (0.865)	−0.151; 0.127	0.009 (0.895)	−0.129; 0.147	−0.127 (0.541)	−0.539; 0.283	−0.140 (0.504)	−0.553; 0.272	2.288 (0.001) *	0.976; 3.599	2.105 (0.002) *	0.808; 3.402
MCHC	0.039 (0.524)	−0.081; 0.159	0.046 (0.459)	−0.077; 0.169	−0.381 (0.035) *	−0.736; 0.027	−0.431 (0.020) *	−0.796; −0.067	1.441 (0.014) *	0.289; 2.593	1.281 (0.031)	0.114; 2.448
PLT	3.533 (0.177)	−1.602; 8.668	2.529 (0.342)	−2.716; 7.774	−0.625 (0.935)	−15.92; 14.67	−5.080 (0.525)	−20.81; 10.65	30.49 (0.228)	−19.20; 80.17	22.70 (0.374)	−27.58; 72.98
RDW	0.028 (0.523)	−0.058; 0.114	0.010 (0.819)	−0.076; 0.097	0.254 (0.048) *	0.001; 0.508	0.260 (0.048) *	0.002; 0.518	−1.204 (0.004) *	−2.023; −0.385	−1.100 (0.008)	−1.920; −0.279

*. Statistical significance (*p* < 0.05); CI. confidence interval. Regression adjusted for age, sex, weight, and height.

## Data Availability

Data is contained within the article or Supplementary Material.

## Supplementary Materials

The following supporting information can be downloaded at: https://www.mdpi.com/article/10.3390/molecules30153227/s1, Table S1: Correlation matrix between metal concentrations and children’s general characteristics. Figure S1: Comparison of mean Pb, Cd, and Se concentrations (A–C) by sex and sampling site. * Indicates statistically significant differences between males and females within the same site (*p* < 0.05). Figure S2: Spearman’s correlation between metal concentrations in hair by site. Regression lines shown for illustrative purposes only. Figure S3: Comparison of Pb, Cd, and Se concentrations by frequency of consumption of various foods. *. Indicates statistically significant differences between consumption groups (*p* < 0.05). Table S2: Spearman correlation matrix between Pb, Cd, and Se concentrations (µg/g) in hair and the frequency of food consumption (meals/week) in the total population. Table S3: Factor loadings for the variables derived from principal component analysis (PCA).

## References

[1] De La Guardia Gutiérrez M.A., Ruvalcaba Ledezma J.C. (2020). La salud y sus determinantes, promoción de la salud y educación sanitaria. J. Negat. No Posit. Results.

[2] Rollin H.B. (2022). Introduction to the special issue of IJERPH entitled “Prenatal exposure to environmental pollutants and other stressors: Impacts on fetal development, birth outcomes, children’s health and beyond”. Int. J. Environ. Res. Public Health.

[3] Al Osman M., Yang F., Massey I.Y. (2019). Exposure routes and health effects of heavy metals on children. Biometals.

[4] Fu Z., Xi S. (2020). The effects of heavy metals on human metabolism. Toxicol. Mech. Methods.

[5] Rashid A., Bhat R.A., Qadri H., Mehmood M.A. (2019). Environmental and socioeconomic factors induced blood lead in children: An investigation from Kashmir, India. Environ. Monit. Assess..

[6] Charkiewicz A.E., Backstrand J.R. (2020). Lead toxicity and pollution in Poland. Int. J. Environ. Res. Public Health.

[7] Zhao B., Zhao J., Zhou S. (2023). Selenium and toxic metals in human hair of the Dashan Region, China: Concentrations, sources, and antagonism effect. Ecotoxicol. Environ. Saf..

[8] Heidari S., Mostafaei S., Razazian N., Rajati M., Saeedi A., Rajati F. (2022). The effect of lead exposure on IQ test scores in children under 12 years: A systematic review and meta-analysis of case-control studies. Syst. Rev..

[9] Alvarez-Ortega N., Caballero-Gallardo K., Olivero-Verbel J. (2017). Low blood lead levels impair intellectual and hematological function in children from Cartagena, Caribbean coast of Colombia. J. Trace Elem. Med. Biol..

[10] Kumar A., Kumar A., Cabral-Pinto M.M.S., Chaturvedi A.K., Shabnam A.A., Subrahmanyam G., Mondal R., Gupta D.K., Malyan S.K., Kumar S.S. (2020). Lead toxicity: Health hazards, influence on food chain, and sustainable remediation approaches. Int. J. Environ. Res. Public Health.

[11] Ramirez Ortega D., González Esquivel D.F., Blanco Ayala T. (2021). Cognitive Impairment induced by lead exposure during lifespan: Mechanisms of lead neurotoxicity. Toxics.

[12] Marianti A., Anies A., Abdurachim H.R.S. (2016). Causality pattern of the blood lead, monoamine oxidase A, and serotonin levels in brass home industry workers chronically exposed to lead. Songklanakarin J. Sci. Technol..

[13] Zink L., Wiseman S., Pyle G.G. (2023). Single and combined effects of cadmium, microplastics, and their mixture on whole-body serotonin and feeding behaviour following chronic exposure and subsequent recovery in the freshwater leech, Nephelopsis obscura. Aquat. Toxicol..

[14] Owumi S.E., Adedara I.A., Otunla M.T., Owoeye O. (2023). Influence of furan and lead co-exposure at environmentally relevant concentrations on neurobehavioral performance, redox-regulatory system and apoptotic responses in rats. Environ. Toxicol. Pharmacol..

[15] Messaoudi I., El Heni J., Hammouda F., Saïd K., Kerkeni A. (2009). Protective effects of selenium, zinc, or their combination on cadmium-induced oxidative stress in rat kidney. Biol. Trace Elem. Res..

[16] Carranza-Lopez L., Alvarez-Ortega N., Caballero-Gallardo K., Gonzalez-Montes A., Olivero-Verbel J. (2021). Biomonitoring of lead exposure in children from two fishing communities at Northern Colombia. Biol. Trace Elem. Res..

[17] Yohannes Y.B., Nakayama S.M., Yabe J. (2022). Glutathione S-transferase gene polymorphisms in association with susceptibility to lead toxicity in lead-and cadmium-exposed children near an abandoned lead-zinc mining area in Kabwe, Zambia. Environ. Sci. Pollut. Res..

[18] Ebrahimi M., Khalili N., Razi S. (2020). Effects of lead and cadmium on the immune system and cancer progression. J. Environ. Health Sci. Eng..

[19] Joseph N., Kolok A.S. (2022). Assessment of pediatric cancer and its relationship to environmental contaminants: An ecological study in Idaho. Geohealth.

[20] Cruz-Esquivel Á., Díez S., Marrugo-Negrete J.L. (2023). Genotoxicity effects in freshwater fish species associated with gold mining activities in tropical aquatic ecosystems. Ecotoxicol. Environ. Saf..

[21] Chaali N., Bravo D., Ouazaa S. (2022). New insights into arsenic and cadmium distribution and origin in paddy soils using electrical resistivity tomography. J. Appl. Geophys..

[22] Galeano-Páez C., Espitia-Pérez P., Jimenez-Vidal L., Pastor-Sierra K., Salcedo-Arteaga S., Hoyos-Giraldo L.S., Gioda A., Saint’pIerre T.D., García S.C., Brango H. (2021). Dietary exposure to mercury and its relation to cytogenetic instability in populations from “La Mojana” region, northern Colombia. Chemosphere.

[23] Olivero-Verbel J., Caballero-Gallardo K., Negrete-Marrugo J. (2011). Relationship between localization of gold mining areas and hair mercury levels in people from Bolivar, north of Colombia. Biol. Trace Elem. Res..

[24] Zheng J., Li M., Tang B. (2021). Levels, spatial distribution, and impact factors of heavy metals in the hair of metropolitan residents in China and human health implications. Environ. Sci. Technol..

[25] González-Álvarez D., Cabrera Jaramillo A., Cadavid Muñoz N., Agudelo-Echavarría D.M., Soto-Ospina A., Arango Ruiz Á. (2023). Total mercury and methylmercury levels in eggs from laying hens in a mining area in Bajo Cauca, Antioquia, Colombia. Emerg. Contam..

[26] Cadavid Muñoz N., González-Álvarez D., Cabrera Jaramillo A., Soto-Ospina A., Arango Ruiz Á. (2023). Toxicological risk in individuals exposed to methylmercury and total mercury through daily-consumed foodstuffs in one of the mining regions of Bajo Cauca, Antioquia, Colombia. Emerg. Contam..

[27] Córdoba-Tovar L., Marrugo-Negrete J., Barón P.A.R., Díez S. (2023). Ecological and human health risk from exposure to contaminated sediments in a tropical river impacted by gold mining in Colombia. Environ. Res..

[28] Marrugo-Negrete J., Verbel J.O., Ceballos E.L., Benitez L.N. (2008). Total mercury and methylmercury concentrations in fish from the Mojana region of Colombia. Environ. Geochem. Health.

[29] Palomares-Bolaños J.C., Caballero-Gallardo K., Olivero-Verbel J. (2025). Hematological parameters and mercury exposure in children living along gold-mining-impacted rivers in the Mojana Region, Colombia. Biol. Trace Elem. Res..

[30] Hashemi M., Rajabi S., Eghbalian M., Suliburska J., Nasab H. (2023). Demographic and anthropometric characteristics and their effect on the concentration of heavy metals (arsenic, lead, chromium, zinc) in children and adolescents. Heliyon.

[31] Örün E., Yalçın S.S., Aykut O. (2022). Lead, mercury, and cadmium levels in breast milk and infant hair in the late period of lactation in Ankara, Turkey. Int. J. Environ. Health Res..

[32] Heng Y.Y., Asad I., Coleman B., Menard L., Benki-Nugent S., Were F.H., Karr C.J., McHenry M.S. (2022). Heavy metals and neurodevelopment of children in low and middle-income countries: A systematic review. PLoS ONE.

[33] Manjarres-Suarez A., de la Rosa J., Gonzalez-Montes A., Galvis-Ballesteros J., Olivero-Verbel J. (2022). Trace elements, peripheral blood film, and gene expression status in adolescents living near an industrial area in the Colombian Caribbean Coastline. J. Expo. Sci. Environ. Epidemiol..

[34] Liu M., Liu R., Wang R., Ba Y., Yu F., Deng Q., Huang H. (2023). Lead-induced neurodevelopmental lesion and epigenetic landscape: Implication in neurological disorders. J. Appl. Toxicol..

[35] Du B., Zhou J., Lu B., Zhang C., Li D., Zhou J., Jiao S., Zhao K., Zhang H. (2020). Environmental and human health risks from cadmium exposure near an active lead-zinc mine and a copper smelter, China. Sci. Total Environ..

[36] Nakaona L., Maseka K.K., Hamilton E.M., Watts M.J. (2020). Using human hair and nails as biomarkers to assess exposure of potentially harmful elements to populations living near mine waste dumps. Environ. Geochem. Health.

[37] Shimo N.A., Salam M.A., Parvin M., Sultan M.Z. (2023). Assessment of selected metals (chromium, lead and cadmium) in the hair of tannery workers at Hemayetpur, Bangladesh. J. Trace Elem. Miner..

[38] Genchi G., Sinicropi M.S., Lauria G., Carocci A., Catalano A. (2020). The effects of cadmium toxicity. Int. J. Environ. Res. Public Health.

[39] Pierron F., Daffe G., Daramy F., Heroin D., Barré A., Bouchez O., Clérendeau C., Romero-Ramirez A., Nikolski M. (2023). Transgenerational endocrine disruptor effects of cadmium in zebrafish and contribution of standing epigenetic variation to adaptation. J. Hazard. Mater..

[40] Allali S., Brousse V., Sacri A.S. (2017). Anemia in children: Prevalence, causes, diagnostic work-up, and long-term consequences. Expert Rev. Hematol..

[41] López-Rodríguez G., Galván M., González-Unzaga M., Hernández Ávila J., Pérez-Labra M. (2017). Blood toxic metals and hemoglobin levels in Mexican children. Environ. Monit. Assess..

[42] Saha S., Dhara K., Chukwuka A.V., Pal P., Saha N.C., Faggio C. (2023). Sub-lethal acute effects of environmental concentrations of inorganic mercury on hematological and biochemical parameters in walking catfish, *Clarias batrachus*. Comp. Biochem. Physiol. Part C Toxicol. Pharmacol..

[43] Bal C., Karakulak U., Gunduzoz M., Ercan M., Tutkun E., Yilmaz O.M. (2016). Evaluation of subclinical inflammation with neutrophil lymphocyte ratio in heavy metal exposure. J. Clin. Anal. Med..

[44] Xiong L., Fan C., Song J., Wan Y., Lin X., Su Z., Qiu J., Wu W., He Z., Wu Y. (2022). Associations of long-term cadmium exposure with peripheral white blood cell subtype counts and indices in residents of cadmium-polluted areas. Chemosphere.

[45] Prasanthi R.J., Devi C.B., Basha D.C., Reddy N.S., Reddy G.R. (2010). Calcium and zinc supplementation protects lead (Pb)-induced perturbations in antioxidant enzymes and lipid peroxidation in developing mouse brain. Int. J. Dev. Neurosci..

[46] Shin C.Y., Choi J.W., Choi M.S., Ryu J.R., Ko K.H., Cheong J.H. (2007). Developmental changes of the activity of monoamine oxidase in pre-and postnatally lead exposed rats. Environ. Toxicol. Pharmacol..

[47] David M., Turi N., Ain Q.U., Rahman H., Jahan S. (2021). Evaluation of environmental effects of heavy metals on biochemical profile and oxidative stress among children at brick kiln sites. Arch. Environ. Occup. Health.

[48] Tsentsevitsky A.N., Petrov A.M. (2021). Synaptic mechanisms of cadmium neurotoxicity. Neural Regen. Res..

[49] Fairweather-Tait S.J., Bao Y., Broadley M.R., Collings R., Ford D., Hesketh J.E., Hurst R. (2011). Selenium in human health and disease. Antioxid. Redox Signal..

[50] Stoffaneller R., Morse N.L. (2015). A review of dietary selenium intake and selenium status in Europe and the Middle East. Nutrients.

[51] Combs G.F. (2015). Biomarkers of selenium status. Nutrients.

[52] Corvalán C., Garmendia M.L., Jones-Smith J., Lutter C.K., Miranda J.J., Pedraza L.S., Popkin B.M., Ramirez-Zea M., Salvo D., Stein A.D. (2017). Nutrition status of children in Latin America. Obes. Rev..

[53] Rayman M.P. (2012). Selenium and human health. Lancet.

[54] Nuttall K.L. (2006). Evaluating selenium poisoning. Ann. Clin. Lab. Sci..

[55] Błażewicz A., Klatka M., Astel A., Korona-Glowniak I., Dolliver W., Szwerc W., Kocjan R. (2015). Serum and urinary selenium levels in obese children: A cross-sectional study. J. Trace Elem. Med. Biol..

[56] Mistry H.D., Williams P.J. (2011). The importance of antioxidant micronutrients in pregnancy. Oxidative Med. Cell. Longev..

[57] Zeng H., Combs G.F. (2008). Selenium as an anticancer nutrient: Roles in cell proliferation and tumor cell invasion. J. Nutrl. Biochem..

[58] Klotz L.O. (2015). Reactive oxygen species as initiators and mediators of cellular signaling processes. Studies on Experimental Toxicology and Pharmacology.

[59] Zwolak I. (2020). The role of selenium in arsenic and cadmium toxicity: An updated review of scientific literature. Biol. Trace Elem. Res..

[60] Batyrova G., Taskozhina G., Umarova G., Umarov Y., Morenko M., Iriskulov B., Kudabayeva K., Bazargaliyev Y. (2025). Unveiling the role of selenium in child development: Impacts on growth, neurodevelopment and immunity. J. Clin. Med..

[61] DANE Censo Nacional De Población Y Vivienda Colombia, 2018. https://www.dane.gov.co/index.php/estadisticas-por-tema/demografia-y-poblacion/censo-nacional-de-poblacion-y-vivenda-2018.

[62] PDEA Plan de Desarrollo 2016–2019. https://www.bolivar.gov.co/archivos/?p=Planes_de_Desarrollo%2FPlan_de_Desarrollo_2016-2019.

[63] PDM Plan de Desarrollo Municipal de Achí, Bolívar, 2020–2023. https://www.achi-bolivar.gov.co/tema/planes/plan-de-desarrollo.

[64] De la Ossa C.A., Ramírez-Giraldo A.F., Arroyo-Alvis K., Marrugo-Negrete J., Díez S. (2023). Neuropsychological effects and cognitive deficits associated with exposure to mercury and arsenic in children and adolescents of the Mojana region, Colombia. Environ. Res..

[65] Manjarres-Suarez A., Olivero-Verbel J. (2020). Hematological parameters and hair mercury levels in adolescents from the Colombian Caribbean. Environ. Sci. Pollut. Res..

[66] Aljumaili O.I., Ewais E.E.D.A., El-Waseif A.A., Suleiman A.A. (2023). Determination of hair lead, iron, and cadmium in a sample of autistic Iraqi children: Environmental risk factors of heavy metals in autism. Mater. Today Proc..

