# Supplementary material for: Hair Levels of Lead, Cadmium, Selenium, and Their Associations with Neurotoxicity and Hematological Biomarkers in Children from the Mojana Region, Colombia
Source: Molecules. 2025 Aug 1;30(15):3227. doi: 10.3390/molecules30153227 (PMC12348745; doi:10.3390/molecules30153227)
Supplement: Supplementary file 1 [file molecules-30-03227-s001.zip › molecules-3721038-supplementary.pdf]

Supplementary Materials

Hair Levels of Lead, Cadmium, Selenium and Their Associations with Neurotoxicity and Hematological Biomarkers in Children from the Mojana Region, Colombia

Jenny Palomares-Bolaños [1,2], Jesus Olivero-Verbel [2] and Karina Caballero-Gallardo [1,2,] *

1 Functional Toxicology Group. School of Pharmaceutical Sciences. Zaragocilla Campus, University of Cartagena, Cartagena 130014, Colombia
2 Environmental and Computational Chemistry Group. School of Pharmaceutical Sciences, Zaragocilla Campus, University of Cartagena, Cartagena, 130014, Colombia
* Correspondence: Author:
Prof. Karina Caballero Gallardo, Ph.D.
Functional Toxicology Group
School of Pharmaceutical Sciences
University of Cartagena
Cartagena, Colombia
Tel: 312-636-3365
Fax: 57-(5)-6699771
E-mail: kcaballerog@unicartagena.edu.co

Table S1. Correlation matrix between metal concentrations and children's general characteristics.

Variable	Hair Pb (µg/g)	Hair Cd (µg/g)	Hair Se (µg/g)	Age (year)	Weight (kg)	Height (cm)
**Hair Pb (µg/g)**	1.000					
**Hair Cd (µg/g)**	**0.172***	1.000				
**Hair Se (µg/g)**	**-0.134***	0.080	1.000			
**Age (year)**	-0.064	-0.027	-0.039	1.000		
**Weight (kg)**	-0.103	-0.054	-0.062	**0.771***	1.000	
**Height (cm)**	**-0.116***	-0.052	-0.003	**0.849***	**0.860***	1.000

The Spearman correlation coefficient is shown. * Statistical significance ($p < 0.05$).

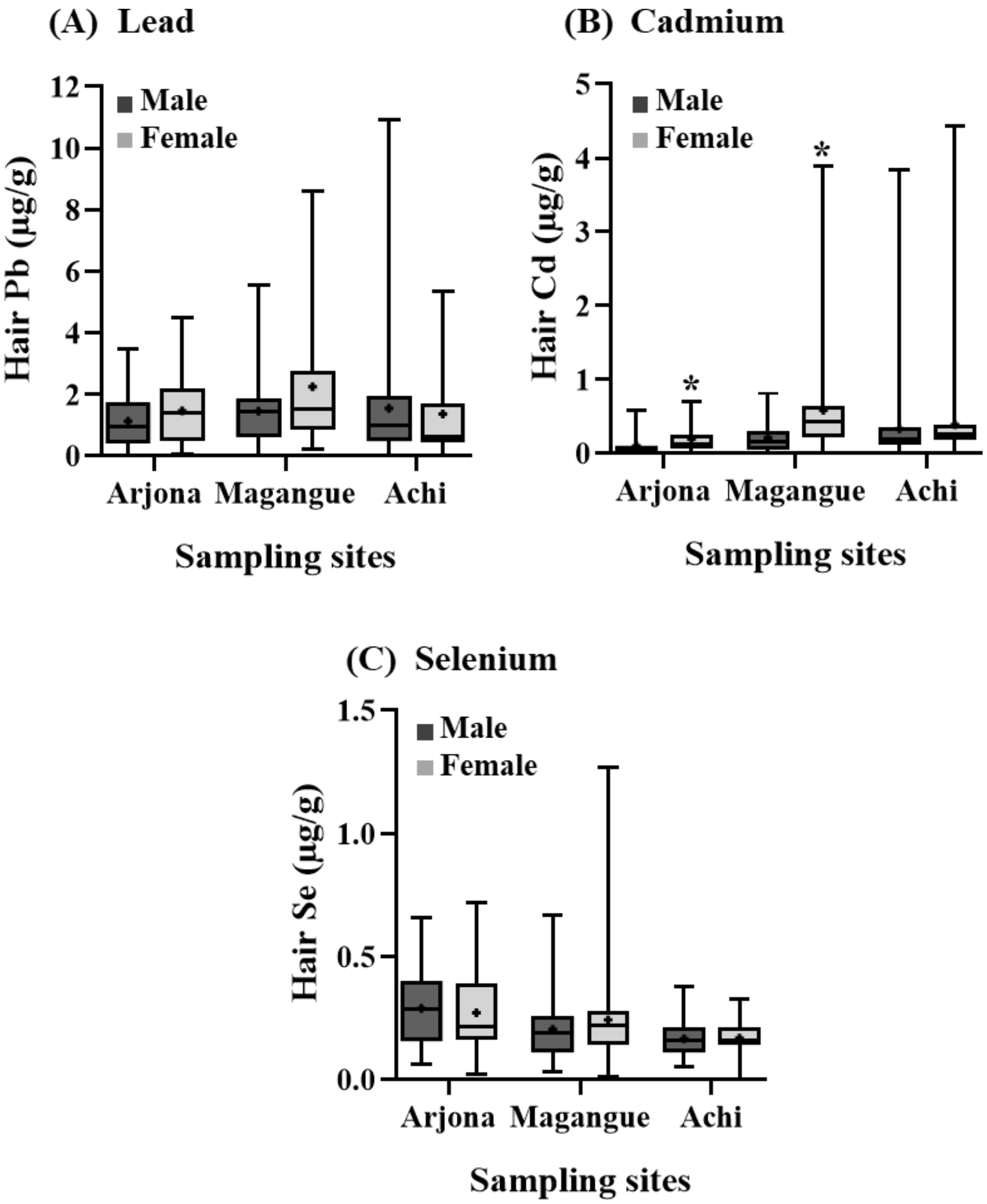

Figure S1. Comparison of mean Pb, Cd, and Se concentrations (A–C) by sex and sampling site. * Indicates statistically significant differences between males and females within the same site (p<0.05).

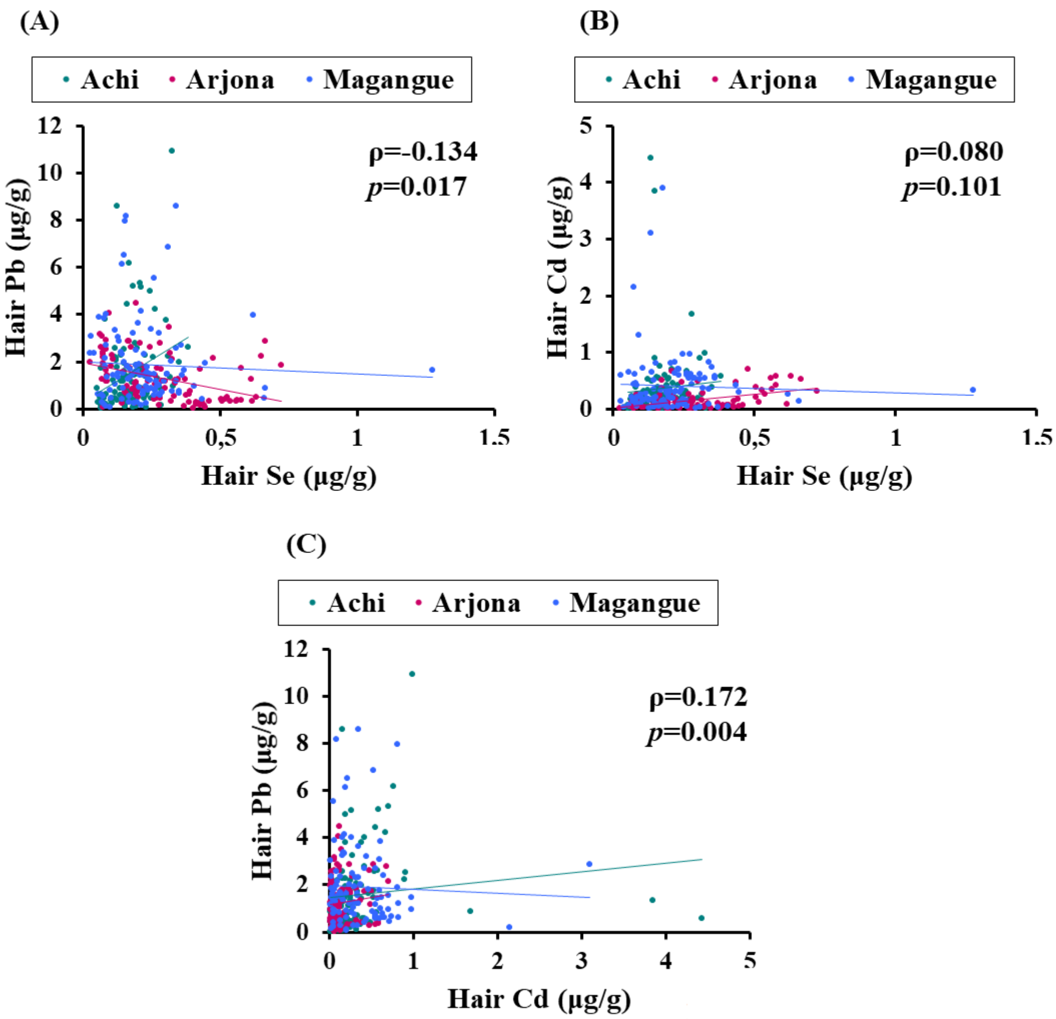

Figure S2. Spearman's correlation between metal concentrations in hair by site. Regression lines shown for illustrative purposes only.

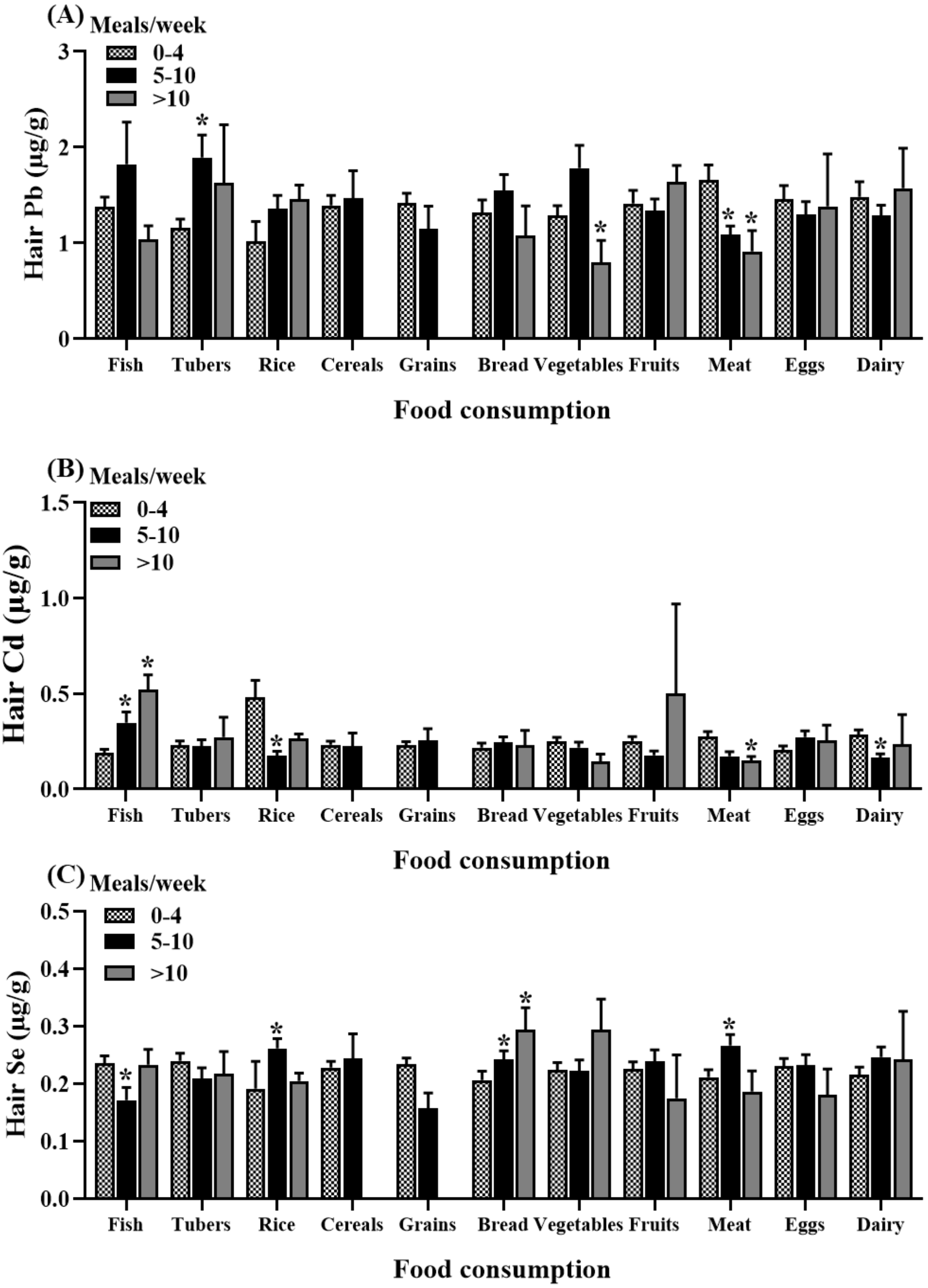

Figure S3. Comparison of Pb, Cd and Se concentrations by frequency of consumption of various foods. *. Indicates statistically significant differences between consumption groups (p<0.05).

Table S2. Spearman correlation matrix between Pb, Cd, and Se concentrations (µg/g) in hair and the frequency of food consumption (meals/week) in the total population.

Variable	Hair Pb (µg/g)	Hair Cd (µg/g)	Hair Se (µg/g)	Fish	Tubers	Rice	Cereals	Grains	Bread	Vegetables	Fruits	Meat	Eggs	Dairy
Hair Pb (µg/g)	**1**		***		**							*		
Hair Cd (µg/g)	0,143	**1**		*		*	*					**		**
Hair Se (µg/g)	**-0,289**	0,050	**1**			**			***					*
Fish	0,051	**0,209**	-0,055	**1**		**		*	***			***		
Tubers	**0,203**	-0,078	-0,069	0,133	**1**		*	**						
Rice	0,072	**0,171**	**-0,223**	**0,204**	0,120	**1**							*	
Cereals	0,007	**-0,175**	0,078	0,058	**0,183**	-0,100	**1**			**	**	***		***
Grains	0,024	0,136	0,142	**0,167**	**0,224**	-0,100	0,094	**1**	***				***	
Bread	-0,001	-0,014	**0,278**	**0,383**	0,137	0,061	0,110	**0,335**	**1**					*
Vegetables	0,048	-0,085	0,059	0,040	0,138	-0,065	**0,211**	0,109	-0,032	**1**				
Fruits	0,092	-0,115	0,009	-0,134	0,103	-0,073	**0,203**	0,033	-0,045	0,104	**1**	***		***
Meat	**-0,170**	**-0,229**	0,144	**-0,275**	0,074	-0,081	**0,285**	0,093	-0,001	0,110	**0,441**	**1**		***
Eggs	0,025	0,080	-0,023	0,033	0,069	**-0,160**	0,003	**0,271**	0,118	-0,041	-0,049	-0,032	**1**	
Dairy	-0,051	**-0,221**	**0,170**	-0,055	0,019	-0,099	**0,326**	0,074	**0,167**	0,125	**0,288**	**0,306**	-0,005	**1**

*Values in bold are different from 0 with a significance level of alpha=0.05.

Table S3. Factor loadings for the variables derived from principal component analysis (PCA).

Variable	F1	F2	F3	F4	F5
Hair Pb (µg/g)	0.115	-0.012	-0.055	0.123	0.271
Hair Cd (µg/g)	0.156	-0.052	-0.132	0.042	0.432
Hair Se (µg/g)	-0.253	0.257	-0.016	0.222	-0.074
WBC	0.635	0.323	0.140	-0.137	0.550
RBC	0.170	-0.423	0.813	0.219	-0.158
HGB	-0.356	0.172	0.775	0.373	-0.032
HTC	-0.047	-0.068	0.846	0.303	0.184
MCV	-0.379	0.498	-0.090	0.079	0.460
MCH	-0.595	0.698	-0.099	0.151	0.097
MCHC	-0.458	0.436	-0.052	0.125	-0.365
PLT	0.379	0.186	-0.185	0.789	-0.077
PCT	0.230	0.327	-0.016	0.593	-0.063
MPV	-0.381	0.198	0.407	-0.572	0.046
PDW	-0.368	0.264	0.352	-0.543	-0.005
RDW	0.635	-0.457	0.038	-0.117	-0.151
LYM	-0.052	-0.217	0.043	0.093	0.810
GRA	0.758	0.486	0.178	-0.165	0.255
LYM %	-0.687	-0.534	-0.127	0.214	0.255
GRA %	0.652	0.573	0.165	-0.142	-0.303
Value	3.727	2.668	2.440	2.120	1.894
Variance (%)	19.617	14.043	12.845	11.156	9.969
% cumulative	19.617	33.660	46.504	57.660	**67.629**
